# Investigation of the Stability of Methylammonium Lead Iodide (MAPbI_3_) Film Doped with Lead Cesium Triiodide (CsPbI_3_) Quantum Dots under an Oxygen Plasma Atmosphere

**DOI:** 10.3390/molecules26092678

**Published:** 2021-05-03

**Authors:** Pao-Hsun Huang, Chi-Wei Wang, Shui-Yang Lien, Kuan-Wei Lee, Na-Fu Wang, Chien-Jung Huang

**Affiliations:** 1 School of Information Engineering, Jimei University, 183 Yinjiang Rd., Jimei District, Xiamen 361021, China; ph.huang@jmu.edu.cn; 2 Department of Applied Physics, National University of Kaohsiung, Kaohsiung University Rd., Kaohsiung 81148, Taiwan; m1074306@mail.nuk.edu.tw; 3 School of Opto-Electronic and Communication Engineering, Xiamen University of Technology, Xiamen 361024, China; 4 Department of Materials Science and Engineering, Da-Yeh University, Dacun, Changhua 51591, Taiwan; 5 Fujian Key Laboratory of Optoelectronic Technology and Devices, Xiamen University of Technology, Xiamen 361024, China; 6 Department of Electronic Engineering, I-Shou University, Kaohsiung 84001, Taiwan; kwlee@isu.edu.tw; 7 Department of Electronic Engineering, Cheng Shiu University, Kaohsiung 84008, Taiwan; k0481@gcloud.csu.edu.tw

**Keywords:** composite perovskite, doped, quantum dots, methylammonium lead iodide, oxygen plasma

## Abstract

In this study, we describe composited perovskite films based on the doping of lead cesium triiodide (CsPbI_3_) quantum dots (QDs) into methylammonium lead iodide (MAPbI_3_). CsPbI_3_ QDs and MAPbI_3_ were prepared by ligand-assisted re-precipitation and solution mixing, respectively. These films were optimized by oxygen plasma treatment, and the effect of powers from 0 to 80 W on the structural properties of the composited perovskite films is discussed. The experimental results showed that the light-harvesting ability of the films was enhanced at 20 W. The formation of the metastable state (lead(II) oxide and lead tetroxide) was demonstrated by peak differentiation-imitating. A low power enhanced the quality of the films due to the removal of organic impurities, whereas a high power caused surface damage in the films owing to the severe degradation of MAPbI_3_.

## 1. Introduction

Over the past several years, the study of inorganic halide perovskite as an optoelectronics material has gained significant consideration because of its extensive waveband (near-infrared) absorption and unique structural properties [1,2,3,4]. Quantum dots (QDs) display advantageous of optical and electrical properties [5,6] via the solution processing function. Therefore, turning inorganic halide perovskite into quantum dots by decreasing particle size to the nanoscale has become a dominant research subject in materials science, even at the commercialization stage. Meanwhile, promising applications in optoelectronics [7,8,9] also involve QDs due to their excellent photophysical properties. At present, the synthesis of QDs is based on various methods, including hot injection, ligand-assisted re-precipitation (LARP), ultrasonication, and solvothermal synthesis. Among these, the LARP method is superior to other methods in terms of the following characteristics: low cost, low processing temperature, simple equipment, and high processing rate. In 2016, supersaturated recrystallization (known as LARP) was firstly reported by Xiaoming Li [10]. It is operated at room temperature, occurs within few seconds, and does not require inert gas and injection. The authors utilized the X-type ligands oleic acid and oleoamine (OAm) to favor the formation of nanoparticles, but the stability of the product was poor and, as a consequence, it could not be modified, purified, and preserved for a long time. Besides, repeated purification or water treatment can easily lose the characteristics of PL [11,12,13]. Therefore, QDs were synthesizes with the L-type ligands, instead of the usual X-type ligands, by using OAm [14]. Although progress in the production of high-quality QDs has been made, practical applications of QDs are still a challenge. So far, the vast majority of scientists use hot injection, but this does involves a complex experimental process. Therefore, the LARP method to synthesize QDs with L-type ligands is preferred over other methods because it does not present the problems linked to X-type ligands. Current studies are mostly using lead (Pb), such as inorganic lead cesium triiodide (CsPbI_3_) and organic methylammonium lead iodide (MAPbI_3_) [1,2,3,4,5,6], due to its high stability and high performance resulting from interactions with organic and inorganic functional groups. The degradation of MAPbI_3_ is improved by doping the inorganic CsPbI_3_, inducing the break of MA bonding. The oxygen plasma treatment is also a common way to remove the organic contaminants onto the film surface and optimize the surface morphology. In addition, there are studies that mention that trace amounts of oxygen are beneficial to MAPbI_3_ films [15,16]. However, excess oxygen ions cause oxidation and ion bombardment [17], leading to a decrease of stability and a severe structural destruction of the film surface. In this article, we describe composite perovskite films based on doping of CsPbI_3_ QDs into MAPbI_3_. This innovative film, proposed as a potential material, is optimized by oxygen plasma treatment at different powers, and formation mechanism as well as its structural properties are investigated.

## 2. Results

**Figure 1:**
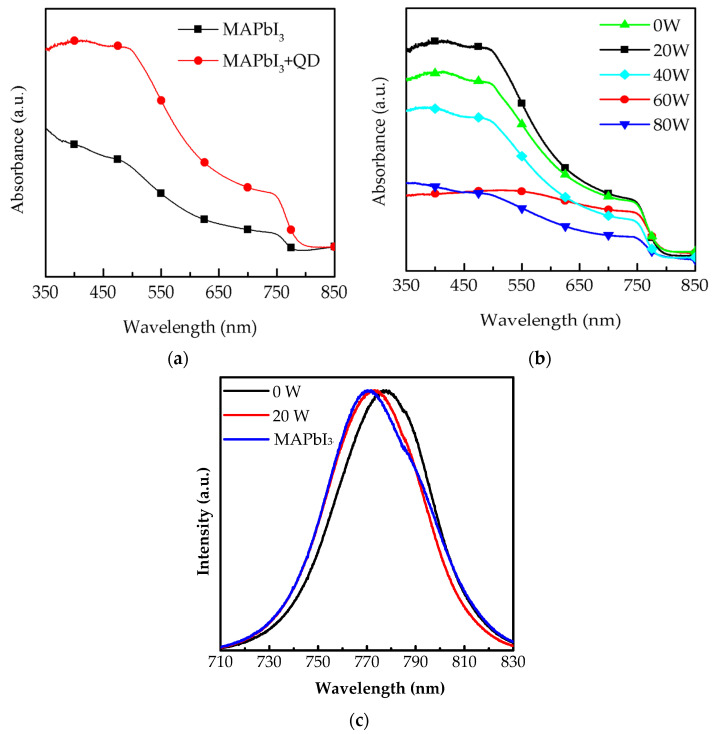
Absorbance spectrum of (**a**) a MAPbI_3_ film with and without CsPbI_3_ QDs spin-coated onto glass substrate and (**b**) further optimization by oxygen plasma treatment from 0 to 80 W. (**c**) Normalized photoluminescence results of the MAPbI_3_ film and composite perovskite films with and without oxygen plasma treatment at 20 W.

The absorbance spectrum of composite perovskite films with MAPbI_3_ and CsPbI_3_ quantum dots (QDs) is shown in the wavelength range from 350 to 850 nm in Figure 1a. Compared to the pure MAPbI_3_ films, composited perovskite films demonstrate obvious absorption bands at 750 nm, proving that their light-harvesting ability in the long-wavelength range is enhanced by doping CsPbI_3_ QDs. The reason is that CsPbI_3_ QDs, a wide-energy gap material, show a small strain at the interface of CsPbI_3_ QDs and MAPbI_3_ [18,19], promoting film growth. Some related studies present the performance of photovoltaic devices, such as solar cells, which are improved after doping quantum dots into perovskite films [20,21]. The surface of composite perovskite films is further optimized via oxygen plasma treatment at different powers, from 20 to 80 W, as shown in Figure 1b. The strongest absorbance of the films was obtained at 20 W due to the removal of the excess impurities on their surface. With power in the range of 40 to 80 W, the absorbance gradually decreased owing to the degradation of MAPbI_3_ induced by the bombardment of oxygen ions at high power [17]. Another possible reason could be that the film surface suffered from the damage of oxygen ion bombardment, and this caused structure dispersion, similar to what observed in studies on plasma engineering [22]. Figure 1c shows the normalized photoluminescence (PL) spectra of the MAPbI_3_ films and composite perovskite films with and without oxygen plasma treatment at 20 W. It is observed that the luminous peak of the composite perovskite films presents an obvious red shift from 770.4 to 776.7 nm due to the doping of CsPbI_3_ QDs. In addition, the sample revealed an obvious blue shift to 773.2 nm after treating with oxygen plasma t 20 W, which may be attributed to the removal of the excess ligand and precursor. We also observed that, the intensity of these two samples was enhanced. These results were further proved by the crystallization measurement indicating variation of orientation.

**Figure 2:**
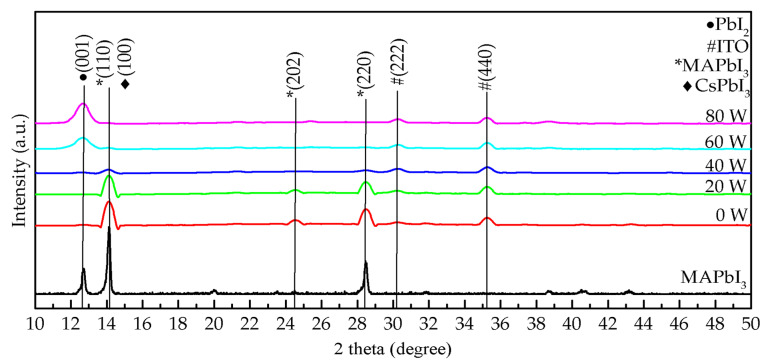
XRD pattern of the composite perovskite films treated with oxygen plasma at various powers from 0 to 80 W.

Figure 2 demonstrates the X-ray diffraction (XRD) pattern of composite perovskite films composed of MAPbI_3_ and CsPbI_3_ QDs and treated with oxygen plasma at different powers, from 0 to 80 W. Based on the spectra of conventional MAPbI_3_ films [23,24], the peak position for MAPbI_3_ in composite perovskite films treated at 0, 20, and 40 W appeared at 14° and 28°. Compared to pure MAPbI_3_ films reported in other studies [23,24], the peak of PbI_2_ easily appeared in their XRD patterns, and PbI_2_ could cause a decrease of the absorption and the formation of defects in MAPbI_3_ films. This phenomenon was not found in our study. However, this phenomenon could be avoided by doping QDs in MAPbI_3_ film. The growth in the (001) orientation of PbI_2_ was not observed in the composite perovskite films. The doping of CsPbI_3_ QDs usefully inhibits the formation of PbI_2_ and even avoids the degradation of MAPbI_3_. This is due to the decrease of hydrogen bonding in MAPbI_3_ and the increase of octahedral tilting by the Cs ion exchange process [25,26]. When the power was higher than 60 W, the peaks of MAPbI_3_ at 14° and 28° disappeared, and then the (001) orientation of PbI_2_ emerged at the peak position of 12°. Generally, oxygen plasma treatment is used to remove impurities or organic materials from a film surface, thus improving it or optimizing the deposition frame. Excess ligand (oleylamine) and precursor (PbI_2_) were removed when the oxygen plasma power was below 20 W. At the power of 40 W, degradation of MAPbI_3_ occurred, and then PbI_2_ was successively produced. When the power increased to 60 and 80 W, oxygen ion bombardment caused severe structural damage or dispersion. This result is similar to those of other studies [17,22]. Thus, high-quality composite perovskite films can be prepared with lower oxygen plasma powers below 20 W.

**Figure 3:**
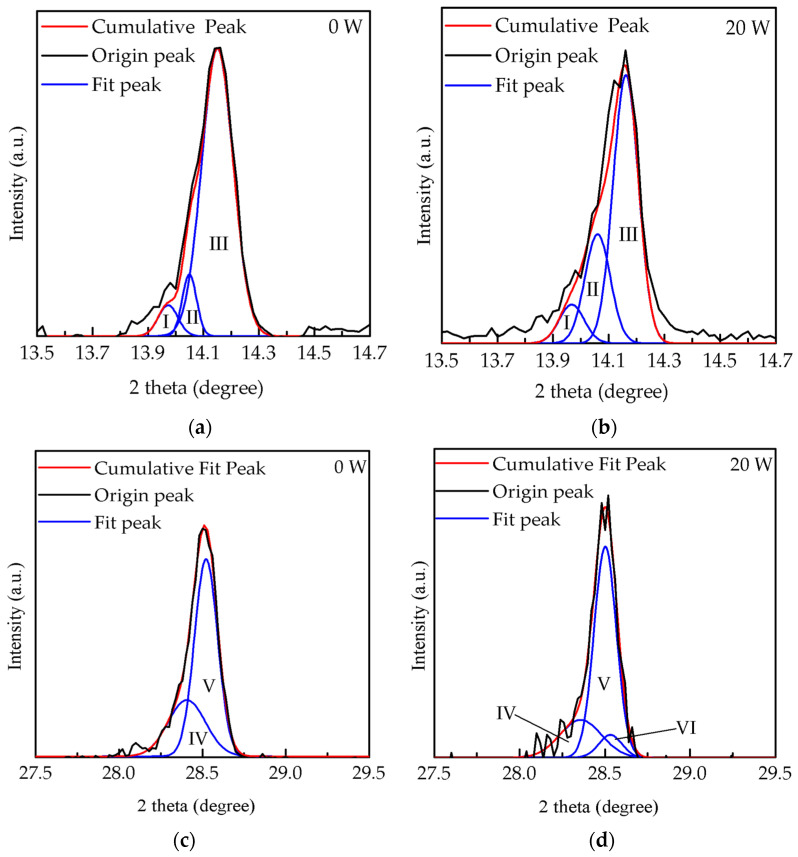
Deconvolution results of films treated at the powers of (**a**) 0 W and (**b**) 20 W in the range from 13.5° to 14.7° and at (**c**) 0 W and (**d**) 20 W in the range from 27.5° to 29.5° through the peak differentiation-imitating.

**Figure 4:**
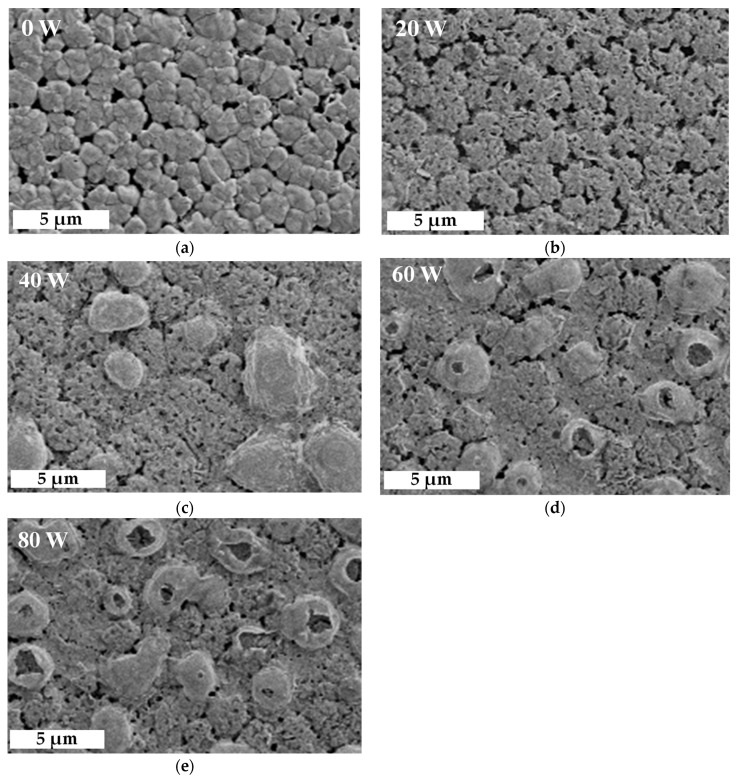
Top-view SEM images of perovskite films composed of MAPbI_3_ and CsPbI_3_ QDs treated at different oxygen plasma powers, from (**a**–**e**) 0–80 W.

As shown in Figure 3, the deconvolution results obtained at 0 and 20 W were analyzed by the peak differentiation-imitating to illustrate the proportion of CsPbI_3_ and MAPbI_3_ peaks which overlapped in the XRD pattern. The peaks of (I), (II), and (III) are represented in the (100) orientation of CsPbI_3_ at 13.97°, the (110) orientation of lead tetroxide (Pb_3_O_4_) at 14.05°, and the (110) orientation of MAPbI_3_ at 14.16°, respectively [27,28,29]. In Figure 3a,b, the peaks (III) and (I) are obviously the major and minor phases, respectively. The area ratios of the peaks (III) and (I) at 0 W were estimated at 85.7% and 8.3% and then varied to 64% and 9% at 20 W, respectively. Notably, when the power increased from 0 to 20 W, the Pb_3_O_4_ proportion dramatically increased from 5.8% to 26%, possibly due to the bonding of lead (Pb) in MAPbI_3_ with oxygen ions [30,31]. Meanwhile, these several oxygen ion species also bound to the carbon in MAPbI_3_, leading to the bonding of carbonyl groups (CO_X_), as reported in other studies [32]. Furthermore, compared to the peak position of MAPbI_3_ films at (110) orientation, the composite perovskite films showed a peak shift of 0.02°, from 14.14° to 14.16°. This result indicated that the exchange of Cs ion to methylamine ion occurred in the MAPbI_3_ film with the increased oxygen plasma power, because the size of the Cs ion is smaller than that of the methylamine ion [33], leading to a decrease of the interplanar distance (*d*-spacing), from 3.153 to 3.148 Å. The *d*-spacing value (*d*) for the (110) orientation of MAPbI_3_ was calculated following Bragg equation [34]:

*d* = nλ/2sinθ (1)

where the n is the order of diffraction, λ is the wavelength of the X-ray sources, and θ as Bragg angle is the peak position of the (110) orientation of MAPbI_3_. On the other hand, in Figure 3c,d, the peaks of (IV) and (V) are represented in the (200) orientation of CsPbI_3_ at 28.4° and in the (220) orientation of MAPbI_3_ at 28.5°, respectively. Another shoulder peak of (VI) at (111) orientation at 28.54° is lead(II) oxide (PbO). The area ratio of the peak (VI) at 20 W decreased from 31% to 22%, mainly owing to the extra bonding of lead (Pb) in MAPbI_3_ with oxygen ions [31,35]. Figure 4 shows top-view SEM images of films treated with oxygen plasma at different powers to further analyze their crystalline growth. Island clusters with obvious grain boundaries are observed initially in Figure 4a. As shown in Figure 4b, when the power increased to 20 W, the oxygen ion induced a slight bombardment, causing blurred grain boundaries and fine pinholes on the surface of the films. The grown crystalline structure at 20 W was still clear, as shown by the enhanced peak intensities in the XRD results. At increased powers, from 40 to 80 W, cell-like perovskite (CLP) appeared, and the grain boundaries became undefined, as shown in Figure 4c–e. The CLP number also increased as the power increased; however, at a power higher than 60 W a clear break of CLP was observed, possibly due to the degradation of MAPbI_3_ induced by the excess oxygen ions, resulting in the swelling and destruction of the film surface associated with CO_X_ [30,35]. These results are similar to those of some studies in surface or interfacial engineering via plasma treatment within chemical vapor disposition and atomic layer deposition [36]. Notably, the influence of oxygen ion bombardment gradually increased with increased power. This was proved by the variation of the films’ thickness.

**Figure 5:**
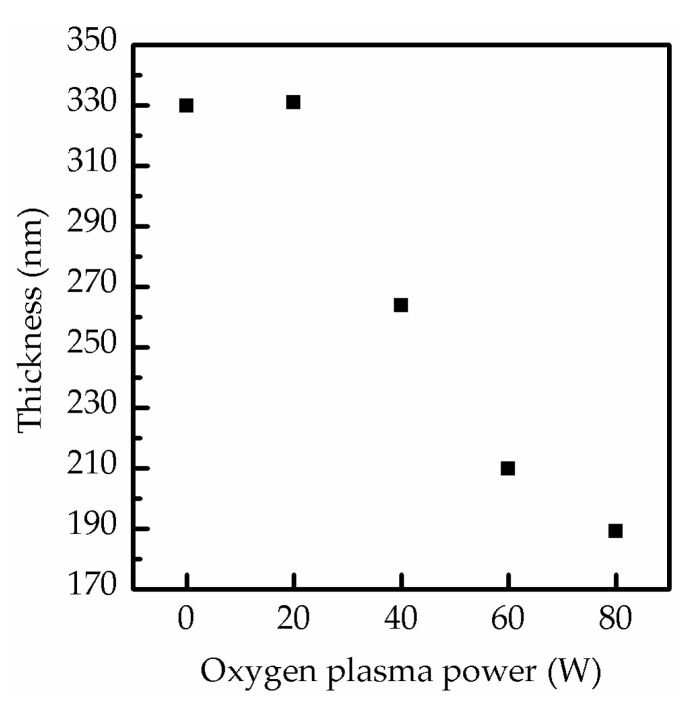
Thickness of perovskite films composed of MAPbI_3_ and CsPbI_3_ QDs after surface treatment at various oxygen plasma powers from 0 to 80 W.

In Figure 5, the thickness of the composite perovskite films is further illustrated at the increase of the oxygen plasma power to demonstrate the influence of oxygen ion bombardment. When the oxygen plasma power was at 0 and 20 W, the film thickness was consistently close to 330 nm. However, the thickness sharply decreased to 263 and 201 nm at 40 and 60 W and then reached the lowest value of 189.3 nm at 80 W. Generally, an increasing plasma power enhances ion bombardment, leading to severe surface damage of the film due to the degradation of MAPbI_3_. The decrease in the degradation rate was evidenced by the decrease of the variation of film thickness (from 20.30% to 6.06%), as shown by the SEM and XRD results. This decreased degradation was due to the saturation reaction of MAPbI_3_ and oxygen [30].

## 3. Materials and Methods

### 3.1. CsPbI3 QDs Fabrication and Centrifugation

**Figure 6:**
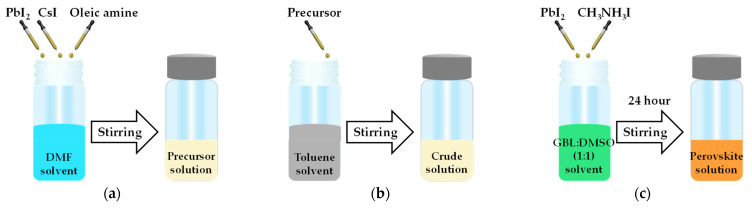
Schematic diagram of the composite perovskite preparation for (**a**) the precursor solution to obtain (**b**) the CsPbI_3_ quantum dots (QDs) solution via the ligand-assisted re-precipitation method (LARP) and (**c**) the MAPbI_3_ solution via solution mixing.

The precursor solution of quantum dots (QDs) was prepared by mixing 0.4 mmol lead iodide (PbI_2_) (Acros organic, 99%) and 0.4 mmol CsI (Alfa Aesar, 99.9%) in oleic amine (2.4 mL) and DMF (J.T. Baker, 99.5%, 10 mL) while continuously stirring for 10 s, as shown in Figure 6a. As shown in Figure 6b, this precursor solution (0.5 mL) was quickly added to toluene (J.T. Baker, 99.8%, 10 mL) while stirring to obtain the CsPbI_3_ QDs solution. After stirring for 10 s, the colloidal crude solution obtained was centrifuged at 11,000 rpm for 15 min at 10 °C. The precipitate was collected and then successively dispersed in hexane. The above process was repeated several times.

As shown in Figure 6c, CH_3_NH_3_I (198.75 mg) and PbI_2_ (576.25 mg) were added into the a mixture of 0.5 mL of sulfoxide (DMSO) and γ–butyrolactone (GBL, 0.5 mL, 1:1 ratio) to obtain the precursor solution [37]. Then, this precursor solution was stirred at 300 rpm for 24 h in a glove box to obtain the perovskite MAPbI_3_ solution.

### 3.2. Fabrication of Composite Perovskite Films

CH_3_NH_3_I (50 μL) and CsPbI_3_ (1 mg) were mixed and then spin-coated onto a glass substrate in two steps, at 1000 rpm for 10 s and 5000 rpm for 20 s. Toluene was dropped on the spinning film for 15 s during the second step. Hereafter, the sample was annealed at 90 °C for 15 min to obtain the composite perovskite films. This composite perovskite films were further enhanced by oxygen plasma treatment at different powers, from 0 to 80 W. The plasma measurement was carried out by RF excitation with a power source of 13.56 MHz (Plasma Etch PC-150 plasma etching/cleaning system).

### 3.3. Characteristics Measurement

The absorbance spectrum of the composited perovskite films was measured by ultraviolet/visible (UV/vis) absorption spectroscopy (HITACHI, U-3900). The X-ray diffraction (XRD) patterns of the films were recorded using a Bruker D8 Discover X-ray diffractmeter with Grazing Incidence X-Ray Diffraction (GIXRD). The top-view surface morphologies of the films were determined by field-emission scanning electron microscopy (FESEM, JEOL-6330). The thickness of the films was estimated by an Ellipsometer (J. A. Woolam/M2000-DI). Normalized photoluminescence (PL) was measured by iHR350.

## 4. Conclusions

In this article, composite perovskite films were successfully prepared by doping CsPbI_3_ QDs into MAPbI_3_. A significant increase in absorbance was obtained at a near-infrared wavelength of 750 nm owing to the doping of CsPbI_3_ QDs. The power of 20 W obviously enhanced the absorbance of the films, possibly owing to the bonding of lead (Pb) in MAPbI_3_ and oxygen ions. According to the SEM images, the surface morphology of the films at 20 W did not suffer excessive damage. However, high powers from 40 to 80 W not only increased ion bombardment causing surface damage of the films but also aggravated the degradation of MAPbI_3_. The proportions of MAPbI_3_, PbO, and Pb_3_O_4_ were further estimated by the peak differentiation-imitating of the XRD results to evaluate the structural properties. The dramatic decrease of MAPbI_3_ from 85.7% to 64% proved that the optimization of composite perovskite films was achieved at the oxygen plasma power of 20 W.

## Data Availability

The data presented in this study are available on request from the corresponding author.
